# Vascular lesions of the pediatric orbit: A radiological walkthrough

**DOI:** 10.3389/fped.2022.734286

**Published:** 2022-11-30

**Authors:** Giovanna Stefania Colafati, Eleonora Piccirilli, Antonio Marrazzo, Alessia Carboni, Andrea Diociaiuti, May El Hachem, Francesco Esposito, Mario Zama, Massimo Rollo, Carlo Gandolfo, Paolo Tomà

**Affiliations:** ^1^ Neuroradiology Unit, Department of Imaging, IRCCS Bambino Gesù Children's Hospital, Rome, Italy; ^2^ Department of Neuroscience, Imaging and Clinical Science, University “G. d'Annunzio” of Chieti, Chieti, Italy; ^3^ Dermatology Unit and Genodermatosis Unit, Genetics and Rare Diseases Research Division, Bambino Gesù Children's Hospital, IRCCS, Rome, Italy; ^4^ Department of Radiology, Santobono-Pausilipon Children Hospital, Naples, Italy; ^5^ Craniofacial Centre-Plastic and Maxillofacial Surgery Unit, IRCCS Bambino Gesù Children's Hospital, Rome, Italy; ^6^ Department of Imaging, IRCCS Bambino Gesù Children's Hospital, Rome, Italy

**Keywords:** vascular malformations, artero-venous malformation, lymphatic malformation (LM), orbital pathology, pediatric orbit, vascular lesion, infantile hemangioma (IH)

## Abstract

Vascular anomalies of the pediatric orbit represent a heterogeneous group that include both vascular tumors and vascular malformations. The disorder may initially be silent and then associated with symptoms and/or function damage, depending on the type of vascular anomaly and its extension. Vascular tumors include benign, locally aggressive (or borderline) and malignant forms while vascular malformations are divided into “simple”, “combined” and syndromic, or “low flow” or “high flow”. Both entities can arise in isolation or as part of syndromes. In this review, we describe the imaging findings of the vascular lesions of the orbit in the pediatric population, which are key to obtain a correct diagnosis and to guide the appropriate treatment in the light of the new genetic and molecular discoveries, and the role of the radiologist in their multidisciplinary management. We will also touch upon the main syndromes associated with orbital vascular abnormalities.

## Introduction and classification

Vascular anomalies of the pediatric orbit represent a heterogeneous group of diseases caused by an abnormal embryological development of vascular structures or by a clonal proliferation of endothelial cells, due to a sporadic or hereditary genetic “error”. This group of pathologies is characterized by (i) morphological and/or hemodynamic alterations, (ii) of blood or lymphatic vessels, (iii) of varying severity and (iv) of any anatomical district (1), including the orbit.

The reported Incidence of vascular orbital lesions in the pediatric population ranges from 5.5% to 22% (2).

They manifest generally at birth or during infancy, with possible progression along life, and a relatively high risk of functional deficit and permanent disfigurement, that hold particularly true for such a small and functionally meaningful anatomical site as the orbit. A multidisciplinary management including clinical features, radiological characteristics, and in some cases, histopathological findings and molecular testing are essential to perform a correct diagnosis in view of an appropriate treatment and follow-up (3, 4). To do so, the use of an appropriate terminology is paramount.

Historically, the inaccurate and controversial terms used in the ophthalmologic literature limited interdisciplinary communication, leading to poor or mismanagement of vascular lesions (5).

These issues have mostly been addressed by the original ISSVA classification and its iterations (2, 6) which still broadly categorize vascular anomalies into vascular tumors (benign, borderline and aggressive) and vascular malformations: by combining histological and radiological features together with the most recent genetic and molecular advances, the latest version of ISSVA (*ISSVA, 2018*. Available online at: issva.org/classification) presents itself as a valid starting point to provide a meaningful and universally accepted classification of vascular anomalies.

However, despite the many efforts, it still has some drawbacks to consider, such as its lack of anatomical specificity. Indeed, it still does not account for some lesions specific to peculiar anatomical sites such as the orbit, while other lesions listed in the classification cannot technically exist in the same location (5).

The aim of this manuscript is to report the main radiological features of orbital vascular anomalies in the pediatric population using the proposed nomenclature of the latest iteration of the ISSVA classification (notwithstanding its limitations in the orbital region). The radiologist’s role is pivotal in the multidisciplinary team for the proper management of these lesions, in order to obtain a correct diagnosis and to guide the appropriate treatment in the light of the new genetic and molecular discoveries.

## Multidisciplinary approach

Successful management of orbital vascular lesions depends on the clinical presentation, site and extension of the lesion, patient age, symptoms and functional and esthetical damage. In addition, since the genetic and molecular background of both inherited and sporadic vascular malformations are currently being elucidated, genetic testing should be performed (i) to confirm the diagnosis in some cases, (ii) in view of a target therapy, and (iii) to perform genetic counselling. Thus, the diagnostic management of a vascular lesion requires an integrated and multidisciplinary approach involving multiple specialists. Radiologists are key part of this team, combining both the diagnostic expertise in imaging and their involvement in their endovascular treatment.

## Imaging strategies and diagnostic work-up

**Figure 1:**
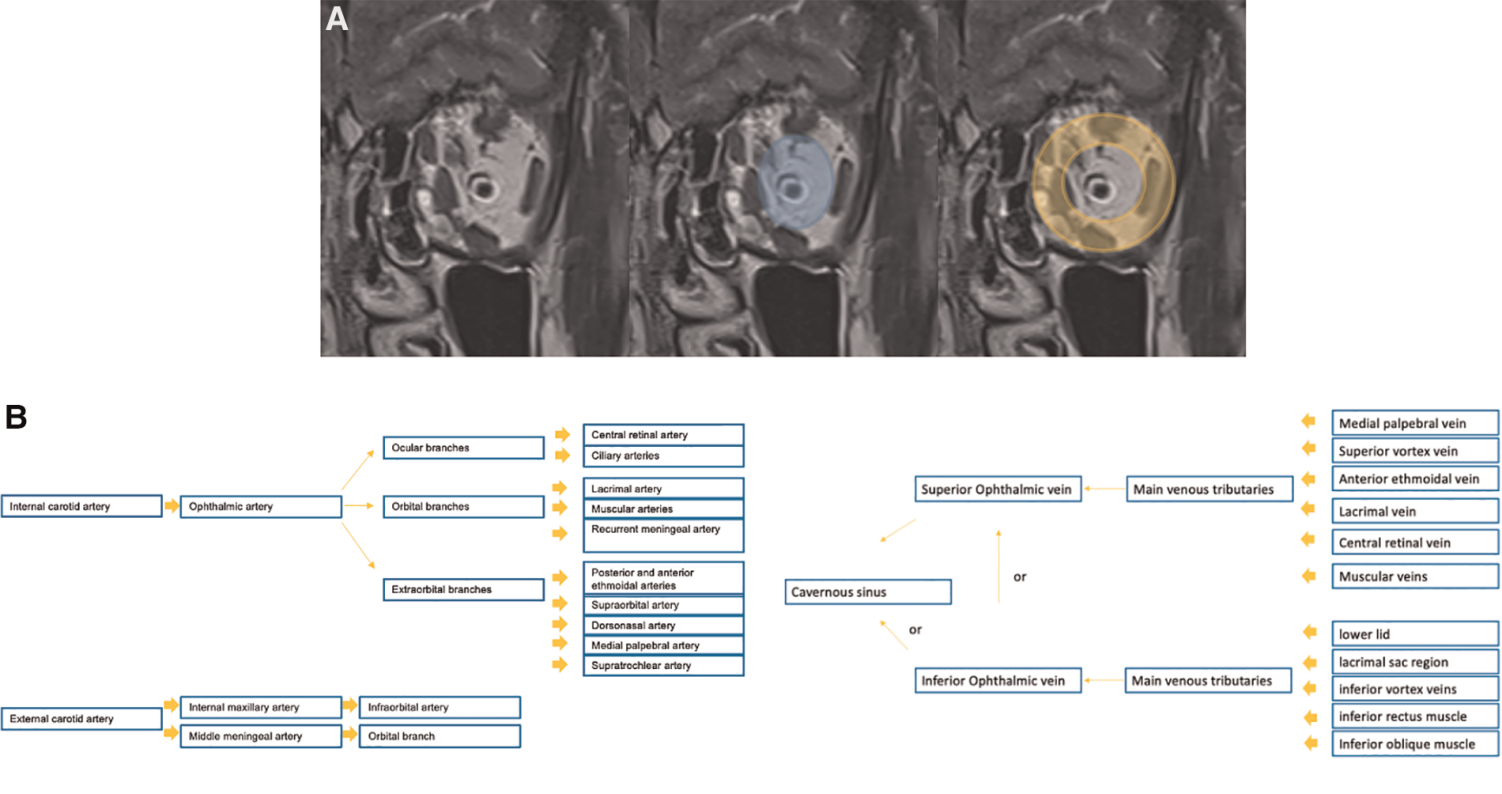
Detailed schemes of the anatomical spaces of the orbit on the coronal T2w sequence (**A**: the intra-conal space is depicted in blue, while the extra-conal in orange) and of the vascular supply of the orbit (**B**).

Imaging evaluation represents an important clue within the diagnostic management of vascular anomalies, and cannot overlook orbital anatomy. For this reason, detailed schemes of the orbital spaces and of the vascular supply of the orbit is provided in Figure 1.

Imaging, other than recognizing the lesion in the first place, contributes to determine lesion extent, organ involvement and potential complications especially in a complex and functionally critical anatomic location as the orbit, and to rule out other entities. Moreover, imaging is paramount for proper treatment planning and for establishing a baseline before treatment.

Mainstay imaging modalities in the pediatric population include Ultrasound (US) and magnetic resonance imaging (MRI), also with angiographic techniques.

**Figure 2:**
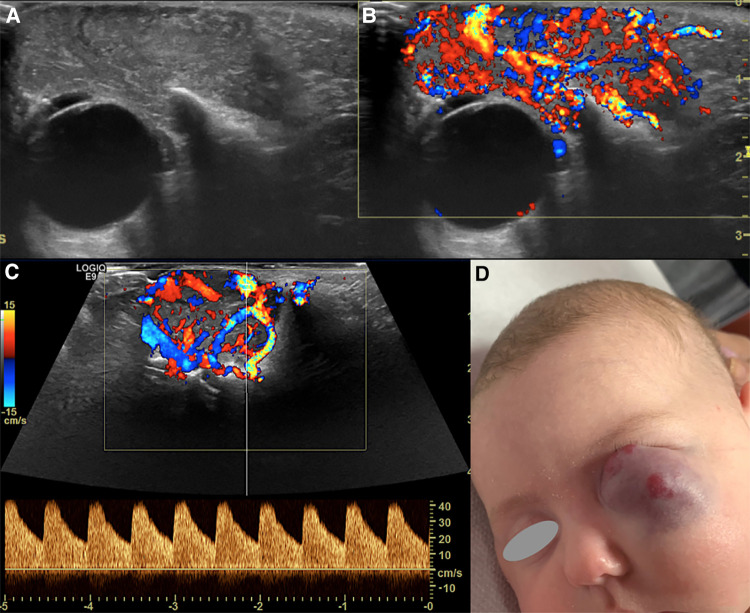
Us of an infantile hemangioma (IH) of the left orbit in a 5-month-old boy, during the proliferative phase. B-mode US shows a hyperechoic mass with well-defined margins overlying the globe (**A**) and a very high vascular density at Color-Doppler US (**B**). Spectral analysis revealed the presence of low resistance arterial flow (**C**). The clinical appearance of the lesion is depicted in (**D**).

The choice of the initial imaging modality varies based on the clinical scenario and specific lesion. Its wide availability, the lack of radiation exposure, little costs and no need for sedation all make US an appropriate initial screening modality after clinical examination in children (7); high-frequency transducers should be preferred, but lower-frequency transducers can be an option in the case of deeper lesions. Grey-scale US delivers an outstanding small parts contrast and assessment of the involved anatomy, and the use of Color Doppler is useful to determine both vascularity and flow dynamics (Figure 2). At US the following features of the lesion must be assessed:

• the anatomical location and the involved layers (e.g., skin, subcutaneous tissue, muscular layer)
• mono vs. multifocality
• the presence of a parenchymal component
• the echogenicity and architecture (presence of fat, calcifications, thrombi, cysts)
• the degree of compressibility
• dynamic changes (i.e. with dependent positions or Valsalva maneuver)
• distribution, density of vascularization, type of vessels (arterial vs. venous) and flow dynamics (high flow vs. low flow) at Doppler study and Spectral analysis
• the status of surrounding tissues
• secondary effects on surrounding structures and organs.

Although it usually follows US in the diagnostic work-up, MRI is the preferred imaging modality due to its higher spatial, contrast and temporal resolution. Particularly, it is obviously indicated (i) in the assessment of large and deeper lesions, (ii) when they are part of complex or syndromic vascular anomalies and/or (iii) prior to invasive treatment. Not secondarily, it allows defining their extension and their anatomic relationship to adjacent structures.

MRI (preferably at high field strengths) can be performed both with head coils (preferably a 32-channel phase-array) and orbital surface coils before and after contrast administration, tailoring the specific protocol to the type of vascular anomaly suspected and its location. The recommended slice thickness is 2–3 mm with a 512 × 512 matrix and no interslice gap. Mandatory sequences should include multiplanar T2 weighted (T2w) fat-suppressed images and pre and post contrast T1 weighted (T1w) images, with or without fat suppression, in at least two orthogonal planes. GRE T2*-weighted images can be added to demonstrate calcification or hemosiderin deposits; on GRE images, generally, low-flow vessels appear as flow-voids, whereas high-flow vessels have high signal intensity. Diffusion Weighted images can be included in the protocol so to help in the differential diagnosis with malignant entities. Contrast–enhanced MR angiography, performed with a 3D T1w fast gradient-echo (GRE) sequence is also needed to evaluate the enhancement pattern of the lesion: usually, imaging is performed in the arterial phase and several venous phases. Images are also obtained before contrast material administration for posterior subtraction of contrast-enhanced images (8). The comprehensive assessment of a vascular lesion must include dynamic time-resolved MR angiography. The high temporal resolution of the sequence – slightly hindered by a relative lower spatial resolution – enables to differentiate high- vs. low-flow malformations and to provide anatomical detail on lesion size and extent. Additional information critical for treatment planning that can be inferred are nidus size, size and number of feeding and draining vessels and connection to a deep venous system (4). Vascular lesions diagnosed in infancy may be part of a syndrome, hence it may be necessary to extend the MRI study to the brain, abdomen, spine and even limbs to look for potential involvement of other body parts. Computer tomography (CT) is usually inadequate: indeed, vascular lesions are visible on CT, but often appear as nonspecific masses. Due to its lower contrast resolution for soft tissues and radiation exposure, CT should be reserved for cases with suspected bone involvement or in the detection of calcifications and whenever MRI cannot be performed. Contrast administration may be more useful for their characterization and the combination dynamic study would allow the mapping of the different arteries and veins.

DSA or venography are mostly used as therapeutic modalities. DSA is most critical for evaluating high-flow vascular malformations (4) and offers a detailed assessment of the vascular anatomy while allowing treatment at the same sitting.

## Vascular tumors

Vascular tumors are formed by the proliferation of vascular tissue and are still divided into benign, locally aggressive and malignant based on their nature (9), with malignant tumors being more commonly encountered in adults. For this reason, among the vascular tumors only the most frequent in the pediatric population will be covered.

### Hemangiomas

Hemangiomas – are the most frequent orbital vascular lesions, taking the “lion’s share” among vascular tumors. The most commonly involved location is the head and neck region (60%) with the orbit being the most frequent (10). Hemangiomas are broadly classified as infantile and congenital.

Infantile hemangiomas (IHs) are usually not visible at birth, but present within the first several weeks of infancy (11). Their appearance can be preceded by a “premonitory cutaneous mark” that looks like a pale spot, telangiectatic or macular red stain, or a bruise-like pseudo-ecchymotic patch (12). During infancy, IHs undergo a rapid growth (phase 1, proliferative) followed by a prolonged period of spontaneous involution (phase 2, involuting) that can last years, during which the lesion is replaced by fat and fibrotic tissue; by late childhood, they have generally involuted completely (phase 3, involuted) (13). IHs are also further divided according to their anatomical configuration into focal, multifocal, segmental and indeterminate (14).

The presence of the immunological marker glucose transporter one (GLUT-1) differentiates IHs from vascular malformations in tissue biopsies (15).

Congenital hemangiomas (CHs) are rare entities (making less than 3% of all hemangiomas) that have long believed not to arise in the orbit. Although even more uncommon, some reports have demonstrated the possibility of CH also arising in this location (16).

CHs are clinically apparent at birth with nearly no growth thereafter (since they have already completed their proliferative phase *in utero*), lack positive staining for GLUT-1 and show *GNAQ/GNA11* genetic changes. In addition, they either involute (more or less rapidly) or never spontaneously involute; according to their involution they are further categorized into Rapidly Involuting Infantile Hemangioma (RICH), Non-Involuting Infantile Hemangioma (NICH) and the newly recognized Partially Involuting Infantile Hemangioma (PICH) (17, 18).

#### Clinical presentation

Their clinical presentation depends on the depth of cutaneous involvement, their size and either the evolutionary phase. Generally, they present as soft masses: visible lesions that are within or near the dermis appear as bright red or pink (“strawberry hemangiomas”), whereas deeper lesions may be blue or purple, therein the name as they have long been known “cavernous hemangiomas” (Figure 2). Complications including ulceration of the tissue, amblyopia (from prolonged unilateral eyelid closure, strabismus or astygmatism, alone or in combination) and stretching of the optic nerve can ensue, ultimately leading to visual loss.

#### Imaging

Although hemangiomas can be suspected clinically in the majority of cases, imaging evaluation is indicated either in the diagnosis of profound hidden hemangiomas to estimate their extension or in masses which are minacious to visual function. The diagnostic workup is primarily based on US. Sometimes MRI (eg in deep lesions) and CT (in a few of cases) are required.

**Figure 3:**
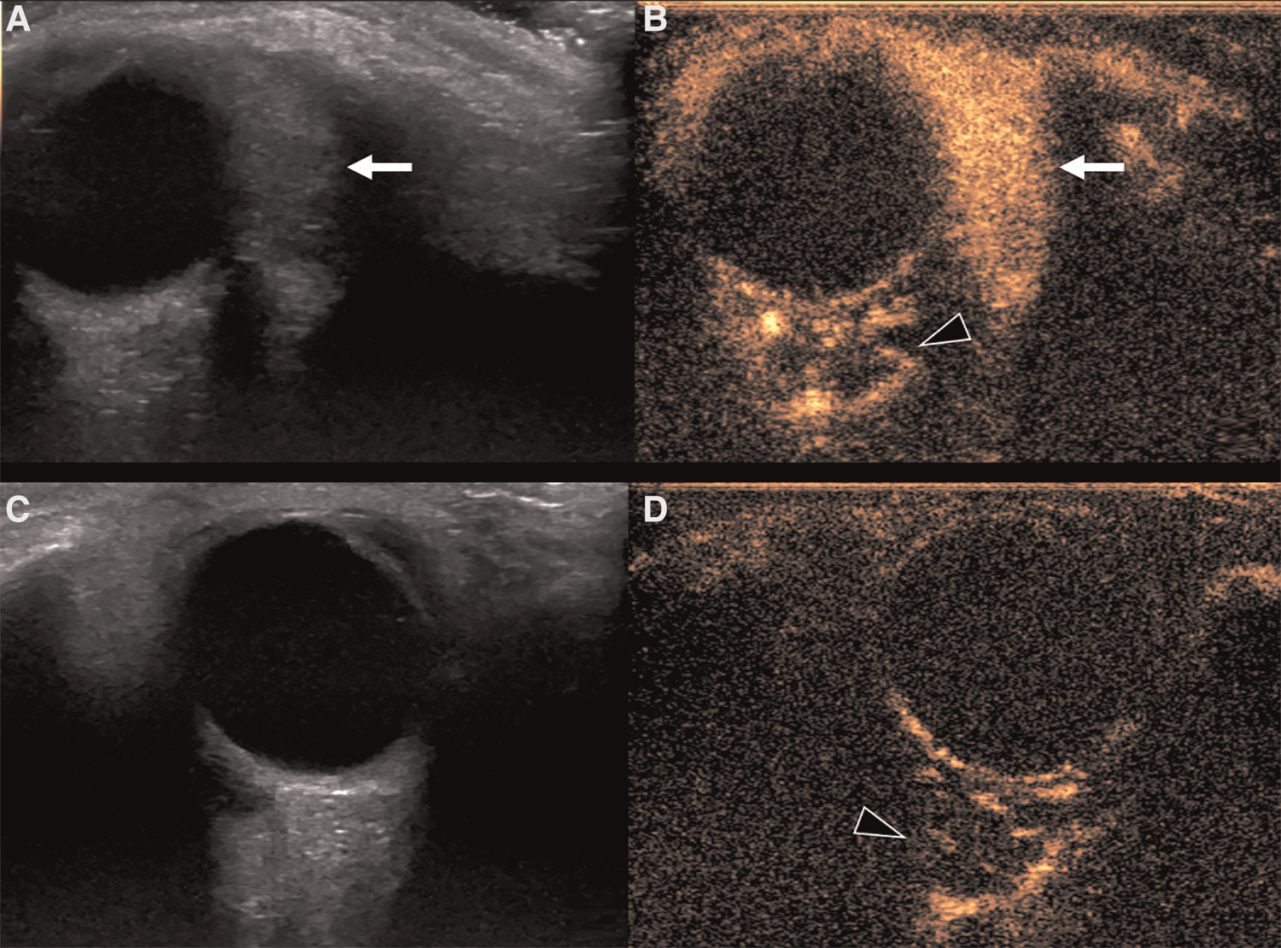
Us and CEUS of a left orbital infantile hemangioma (IH) in a 3-year-old girl: the patient was initially referred for a forehead red macule on the left and a slight ipsilateral orbital proptosis. (**A,B**) Depict US of the left orbit, while US of the right orbit is represented in (**C,D**). At B mode US (**A**) a uniformly hyperechoic mass was discovered (white arrow) with rapid and complete enhancement at CEUS (**B**, white arrow) Of note, left retrobulbar vessels appeared more prominent than the contralateral side (arrowhead in **B** and **D**, respectively). The orbital mass showed the same US characteristics of the IH of the forehead (not shown).

Despite the different pathogenesis, clinical presentation and genetics, CH and IH have almost the same imaging characteristics, particularly during the proliferative phase. However, CHs are more likely to have aneurysms, larger venous components, intravascular thrombi and even high flow velocity at birth, compared to their infantile counterpart (19), ancillary findings that must be carefully be searched for. US demonstrates a well-demarcated tumor with variable echogenicity and high vascularity, showing more than 5 vessels/cm with high flow velocities at Color Flow Doppler during the proliferating phase (20) (Figure 2). During the involuting and involuted phases, flow velocity and vessel density will decrease. Due to its considerable abilities in the assessment of intra-lesional blood flow, CEUS is making its way as a powerful additional tool in the assessment of orbital space-occupying lesions (21, 22). At CEUS hemangiomas are hyper-enhancing during both the early and late arterial phase. Enhancement of IHs starts as a peripheral nodules and then progressively expands in a centripetal pattern and completely fills the lesion (Figure 3). During the delayed phase, hemangiomas keep showing sustained enhancement relative to surrounding tissues.

**Figure 4:**
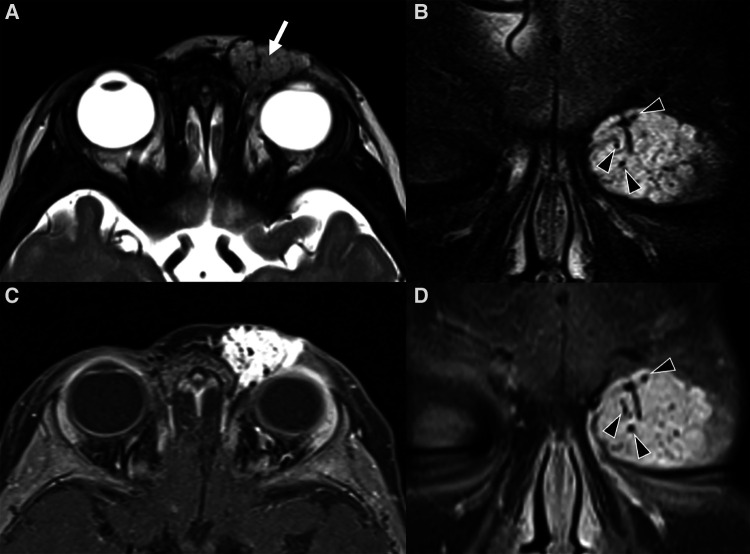
Mr images of a non-syndromic left periorbital infantile hemangioma in a 1-year-old girl. Axial (**A**) and coronal fat-saturated (**B**) T2 weight images show a well-defined hyperintense mass (arrow in **A**) with multiple internal flow voids (black arrowheads in **B** and **D**), extending from the anterior periorbital soft tissues into the extra and intraconal compartments of the orbit. Axial (**C**) and coronal (**D**) contrast-enhanced fat-saturated T1 weighted images show vivid homogeneous contrast enhancement of the vascular lesion.

**Figure 5:**
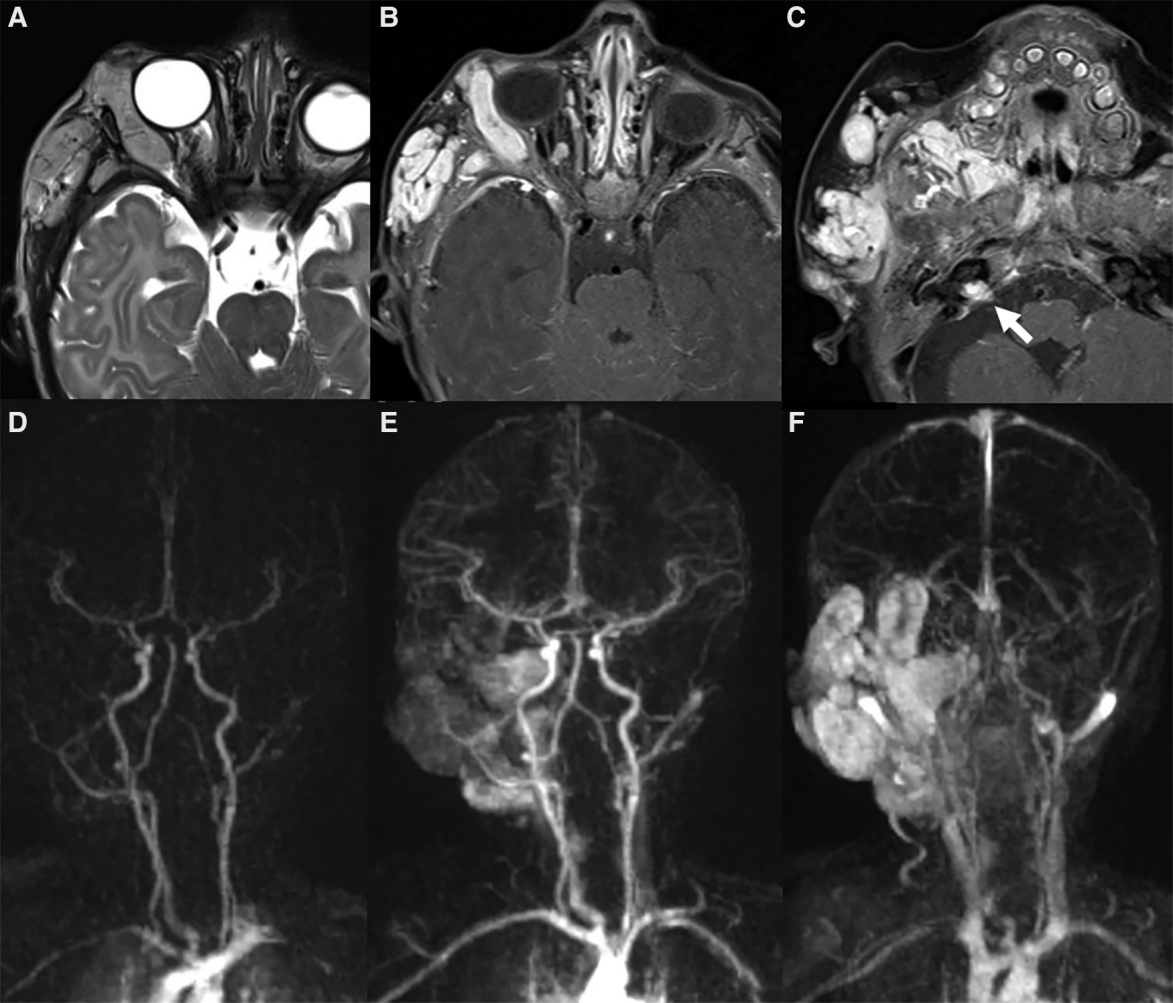
Mr images of a 6-month-old girl with known PHACES. Axial T2 (**A**) and contrast-enhanced T1 weighted images (**B,C**) show a diffuse segmental hemangioma of the right orbital, malar and auricular regions. Coronal dynamic MR angiography during gadolinium injection (**D–F**) shows the “progressive filling” of the vascular lesion. Also note in (**C**) the small enhancing mass in the right internal auditory canal (white arrow), consistent with an intracranial hemangioma, and the ipsilateral cerebellar hypoplasia.

On the other hand, it is MRI that provides the best insights on the architecture and the best accuracy on the extension and spatial relationships of the lesion. Indeed, it may show the tumor involving and enlarging the eyelid and obstructing vision, displacing or distorting the globe or the extra- ocular muscles. On T2w images hemangiomas appear as a well-defined, lobulated and hyperintense masses with flow-voids and thin, dark septa between lobules (Figure 4). These features are even more striking on STIR images (Figure 4). Flow voids are less likely seen in the involuting and involuted phase. On T1w sequences they show a heterogeneous low/intermediate signal with a variable amount of hyperintensity related to fat tissue deposits according to the evolutionary phase. Contrast enhancement is usually homogeneous and intense during the proliferative phase (Figures 4, 5), although it may become less homogenous, intense and predictable in the involuting and involuted phase.

CT is rarely indicated. However, it is especially useful in (i) suspected skeletal involvement, (ii) contraindication to sedation in pediatric patients, (iii) situations requiring urgent imaging, and (iv) view of surgical treatment in some conditions. At CT, proliferating IHs appear as well-demarcated masses that are isoattenuating to muscle and demonstrate early, uniform, intense enhancement with intravenous contrast. Calcifications are rarely seen. In involuting and involuted IHs fat deposition increases, and enhancement becomes less homogeneous. In case of rapidly growing lesions, CT may demonstrate bony orbital expansion or scalloping.

Although the majority of IHs represent an isolated finding, at least 20% of children will have multifocal lesions. In the case of more than 5 IHs (in any body part), the child should be screened with an abdominal US to rule out hepatic hemangiomas (9). Moreover, certain subtypes such as the segmental IH - defined as covering an anatomic territory of the face (or body) – require special management. Indeed, the importance of recognizing a segmental hemangioma in children is twofold.

First off, they are more prone to complications, such as ulceration.

Secondly, segmental IHs are the hallmark of **PHACES** syndrome: PHACES is present in up to 2% of children with facial hemangiomas and 20% of children with “segmental” facial hemangiomas (23).

**Figure 6:**
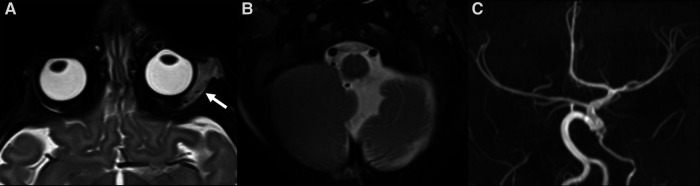
MRI of 4-month-old girl with a red mass of the left orbit. Axial T2 weighted images (**A,B**) show a left orbital hyperintense mass (big white arrow in **A**) with tiny flow-voids involving the extra and intraconal compartments, consistent with infantile hemangioma (IH); on the same sequence the cerebellar atrophy on the ipsilateral side of the IH is also noted (**B**). Additionally, MR TOF-3D disclosed abnormalities of the intracranial circulation (**C**). The diagnosis of PHACES was finally made.

It includes: **P**osterior fossa abnormalities (Dandy-Walker or cerebellar hypoplasia usually on the same side of the hemangioma) (Figures 5, 6), **H**emangioma, **A**rterial abnormalities (Figure 6), **C**ardiac anomalies (coarctation of the aorta), **E**ye defects and **S**ternal abnormalities (24). Importantly, cerebral arterial abnormalities are the most frequently associated abnormalities in the literature (25) and they are usually located on the same side of the IH and cerebellar findings, supporting the embryological pathogenesis (26). Hence, all patients with suspected PHACE should undergo MRI of the brain and of the cerebral vasculature and should be risk-stratified for acute ischemic stroke (25), although strong evidence for the latter are still lacking.

Lastly, the finding of a segmental orbital or, generally facial, IH should prompt a multidisciplinary evaluation in all infants (27).

In the case of orbital location, large intra-orbital hemangiomas with retro-orbital extension have been reported. Moreover, intracranial locations of the hemangiomas, with a keen predilection for the cerebellopontine angle, have also been described (28) (Figure 5).

### Less frequent tumors: tufted angioma (Ta) and kaposiform hemangioendothelioma (KHE)

The rare tufted angiomas (TAs) and kaposiform hendoteliomas (KHEs) have been sporadically reported in the infantile orbit (29).

They share a common genetic background, as tumors somatic mutations of *GNA14* lead to the constitutional activation of MAPK pathway in both (30).

Most frequently, lesions are initially seen as solitary tumors or infiltrating plaques that are dusky red or violaceous, sometimes associated with hyperhidrosis or hypertrichosis (31–33). Typical of the extremities, one-fifth appear in the head and neck area (34), where the sites of involvement include the eyelids, auricle, lips, and oral mucosa (29, 35). They both display a locally aggressive-borderline behavior, are associated with a systemic complication known as Kasabach–Merritt phenomenon (36) and share the same clinical, histological and genetic features: these observation lead to the hypothesis that TAs and KHEs are part of the same clinical spectrum of vascular tumors rather than distinct entities (37).

#### Imaging

Clinical diagnosis of TA is not frequently easy and imaging could be helpful in rare cases: on US, TAs appear as superficial, heterogeneously echoic lesions with ill-defined margins, not exceeding 1 cm in thickness and surrounded by hyperechoic subcutaneous fat (19). However, histopathology on a biopsy on affected tissue is required to define the diagnosis of the lesion. Similarly to TAs, on US, KHAs show heterogeneous echogenicity with ill-defined margins, making them difficult to differentiate from adjacent soft tissues and the diagnosis requires a biopsy (15). MRI is also necessary due to the deep infiltrating nature of KHE which cannot be completely evident on physical exam or US (38).

## Vascular malformations

As per the ISSVA criteria, the term “vascular malformation” should be reserved for lesions resulting from abnormal vascular morphogenesis during the retiform stage of embryogenesis (39) that are present at birth, grow proportionately with the child, do not spontaneously regress and have normal endothelial turnover (40). Unlike vascular neoplasms, vascular malformations do not undergo a proliferative phase. However, they can sensibly increase in size due to hormonal changes during puberty or pregnancy or as a consequence of thrombosis, infection, trauma or incomplete treatment. In most instances these are caused by sporadic somatic gene mutations in tyrosine kinase receptor pathways responsive to vascular endothelial growth factor (VEGF), such as *RAS*, *PIK3CA* and *AKT* (41, 42). In the orbit, vascular malformations are often multi-compartmental, involving pre- and post-septal regions as well as the intra- and extra-conal portions (10). Vascular malformations include (i) *superficial lesions* that typically involve the conjunctiva or lid alone, (ii) *deep lesions* that usually have no surface manifestations and are entirely retrobulbar, (iii) *combined lesions* that have both superficial and deep components and (iv) *complex lesions* that involve the orbit and peri- orbital and intracranial tissues, and may be seen in multifocal sites (43). Vascular malformations can affect facial bones in two different ways: either they may cause a disharmonious overgrowth of the adjoining bone when centered in soft tissues or they can primarily arise within the bone. In any case, bone involvement is better assessed with CT in the diagnostic evaluation.

### Low-flow vascular malformations

Low-flow vascular malformations have no arterial component and are further characterized by their predominant endothelial cell type: capillary, venous, lymphatic or combined (eg, capillary-lymphatic venous malformation, lympho-venous malformation).

### Capillary malformations

#### Clinical presentation

**Figure 7:**
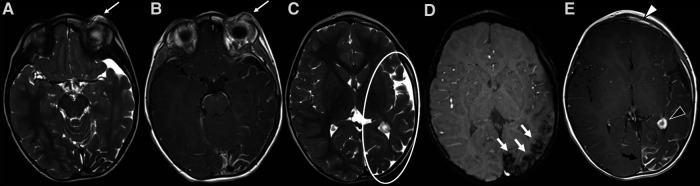
MRI of a 3-year-old child with Sturge-Weber syndrome and a capillary malformation (CM) on the left forehead, upper eyelid and periorbital region (V1 distribution). (**A,B**) On the left there is an increased thickness of the periorbital soft tissues (particularly of the skin and subcutaneous tissue) corresponding to the clinically visible mark, which shows a subtle hyperintensity on axial T2-weighted sequence and a faint but rather homogeneous enhancement on axial post contrast T1 weighted sequences (white arrows in A and B, respectively). (**C–E**) Typical associated intracranial features of SWS on the same side (left) of the CM, including atrophy of the cerebral hemisphere with enlargement of subarachnoid spaces (especially in the frontal, insular, parietal and occipital regions, white circle in **C**), cortical gyral hypointensities due to calcifications on SWI (white arrow in **D**), contrast enhancement of the leptomeningeal angioma and choroidal plexus hypertrophy on axial post-Gadolinium T1 weighted sequence (black arrow and arrowhead in **E**, respectively). A subtle enhancement of the left frontal bone is also evident (white arrowhead in **E**), due to the intra-osseus extension of the overlying CM.

**Figure 8:**
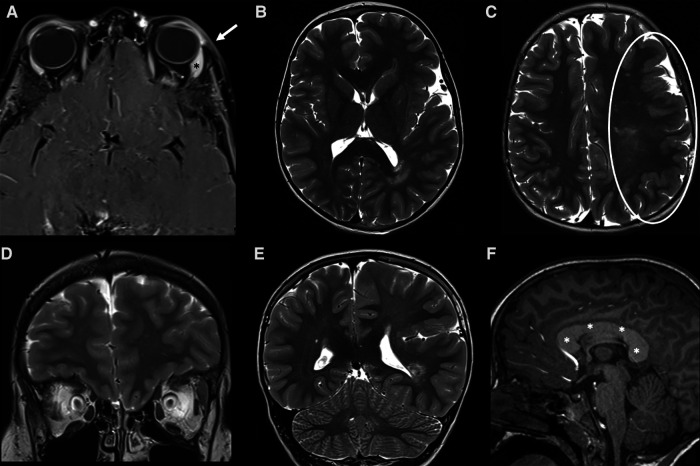
MRI of a 7-year-old Male child with left-sided facial hypertrophy with multiple reddish macules on the face and left orbit, with a clinical suspicion of macrocephaly-capillary malformation (M-CM). (**A**) A slight thickening associated with subtle enhancement of the skin and subcutaneous tissue (white arrow) of the left orbit is noted on fat-sat post-contrast T1 images, corresponding to one of the clinically visible macules. A slight hypertrophy of the left lacrimal gland is also noted (asterisk). (**B–E**) Axial (**B,C**) and coronal (**D,E**) images of the brain revealed a marked asymmetry of the cerebral hemispheres, with left being larger than right consistent with left hemimegalencephaly. The cortex of the enlarged hemisphere is also diffusely polymicrogyric (white circle in **C**). (**F**) on the post contrast sagittal T1 an abnormal thickness of the entire corpus callosum is also evident (white asterisks). A mosaic mutation of PIK3CA gene was discovered, confirming the clinical suspicion of M-CM.

Capillary malformations (CMs) are superficial cutaneous lesions that consist of very small, dilated capillary-like channels and manifest clinically at birth as “port wine stain” macules (34). In the head and neck, the typical anatomic locations include the forehead, eyelids, nose, cheeks and neck (44). Sturge Weber syndrome (SWS, Figure 7) is the neurocutaneous disorder that is classically associated with facial CM, glaucoma and leptomeningeal angioma in its complete form. The finding of CM on the frontonasal placode in a child should prompt ophthalmologic examination and contrast-enhanced MRI that is recommended when the child is 1 year old (45) Mosaic mutations in GNAQ gene has been found to cause both Sturge-Weber syndrome (SWS) and isolated CMs (46). More recently mosaicisms in *GNA11* have also been identified as a cause of both sporadic and syndromic CMs (47, 48). These genetic abnormalities may cause vascular MAPK and/or PI3K signaling pathways alteration (49, 50). Although they have been historically considered the hallmark of SWS, CMs are also the cardinal cutaneous manifestations of other less frequent and known syndromes, such as Capillary Malformation-Arteriovenous Malformation Syndrome (CM-AVM) (51) and Parkes-Weber syndrome, caused by mutations of *RASA1* gene and less commonly *EPHB4* gene (54), Macrocephaly-Capillary Malformation Syndrome (M-CM, Figure 8) and Klippel-Trenaunay syndrome (KTS) caused by mutations of *PIK3CA,* and Proteus syndrome.

#### Imaging

CMs are superficial, therefore their imaging findings - when present - are often subtle and not specific: on US, they are usually isoechoic and confined to the dermis, showing occasionally Doppler flow (34). On MRI they may appear as subtle signal intensity abnormalities within the cutaneous or subcutaneous tissues or simply as skin thickening (13) that can enhance after contrast administration (Figures 7, 8). If imaging is of limited usefulness in their diagnosis, however it does play a major role in ruling out the presence of associated deeper vascular anomalies when a syndromic association is suspected – SWS being by far the most common. While most children with facial capillary malformations do not have Sturge-Weber syndrome, the overall risk of the syndrome in children with vascular malformations of the face - especially those following frontonasal placode - is approximately 8% (52). Due to the severity of the disease, all neonates with facial capillary malformations involving the forehead, eyelids and parietal region or frontonasal area should undergo contrast-enhanced MRI to evaluate leptomeningeal capillary and venular malformations (13) (Figure 7).

### Venous malformations

Venous Malformations (VMs) are composed of an abnormal venous network of varying size and the most common low-flow vascular malformations (53). They can be found everywhere but about 40% of VMs arise in the head and neck (54): common sites include the face, deep neck spaces and orbital region (11). In the orbital region, these malformations can be superficial (anterior, visible lesions limited to the conjunctiva or eyelid), deep (retrobulbar or peribulbar lesions without surface manifestation), combined (superficial and deep), and complex (not only involving the orbit but also the periorbital and intracranial tissues; possibly multifocal and systemic) (55).

#### Clinical presentation

Superficial lesions present as a bluish skin discoloration and are soft, compressible and non-pulsatile lesions that gradually refill when pressure is released (56) while deep lesions may present as lumps with normal overlying skin. VMs typically enlarge during exertion or when in dependent positions and may exhibit spontaneous thrombosis. Less commonly, VMs may present with enophthalmos due to the enlargement of the orbital bone and atrophy of intra-orbital fat (57).

#### Imaging

**Figure 9:**
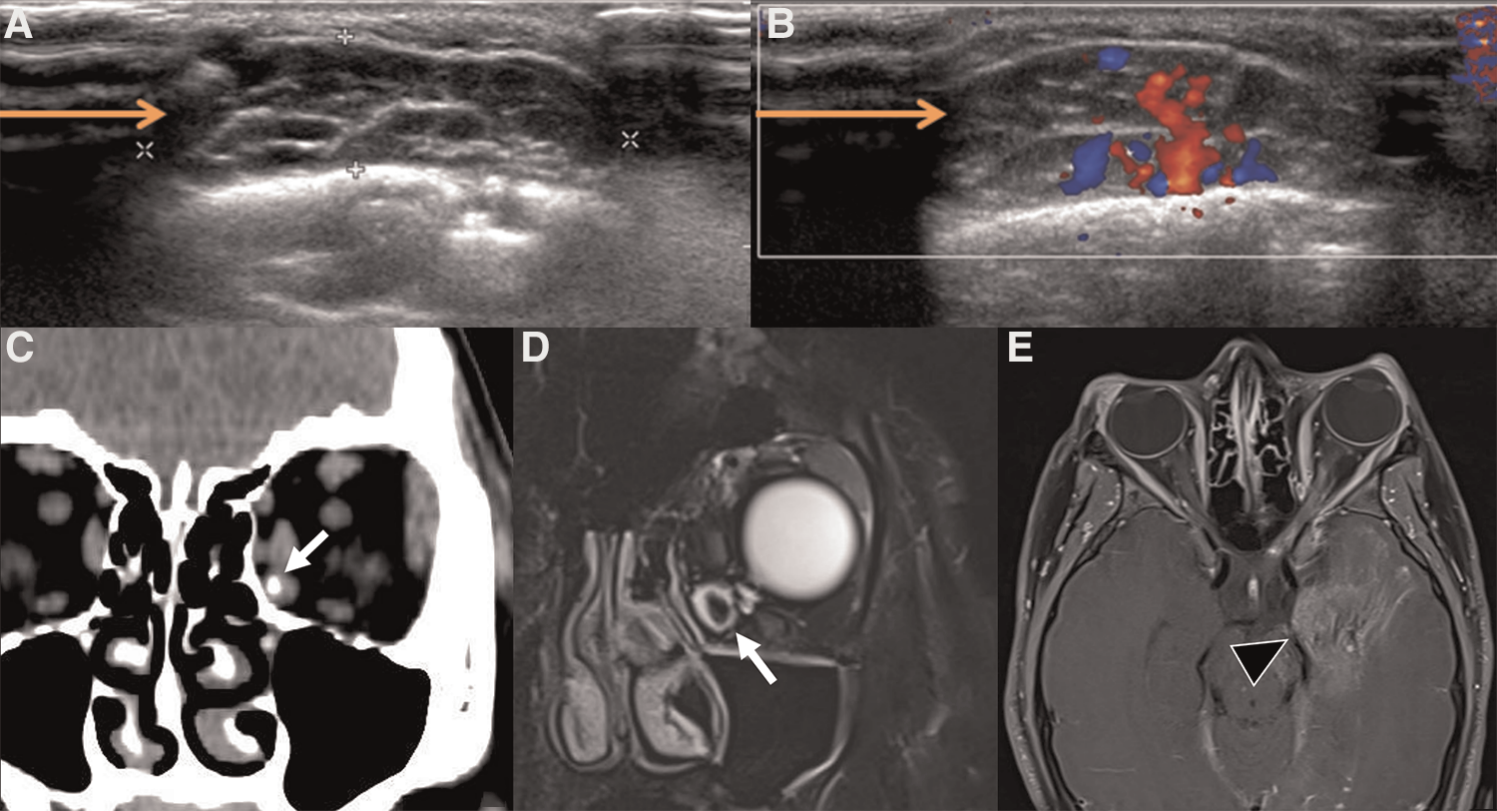
Imaging of venous malformations. (**A,B**) US of a venous malformation of the lid in a 7-year-old boy. B-mode US (**A**) shows a compressible, relatively well-marginated mass with a “spongiform appearance” consisting of multiple anechoic dilated venous spaces separated by hyperechoic septa; on color Doppler US during Valsalva maneuver (**B**) a slight increase in size and increased representation of color-Doppler signal (baseline not shown) can be appreciated. (**C–E**) Coronal CT (**C**), coronal fat-suppressed T2-weighted image (**D**) and axial post-contrast fat-suppressed T1-weighted images (**E**) of a venous malformation of the orbit in a 13-year-old girl. A phlebolith within dilated venous channels is clearly evident at CT and MRI (target sign, white arrows in **C,D**); axial post-contrast FS T1 weighted image also shows an associated diffuse hemispheric venous drainage anomaly (black arrowhead in **E**).

On US, venous malformations often present as compressible, well-marginated masses with a spongiform appearance from the presence of hypoechoic ectatic venous spaces separated by hyperechoic septa; they are generally heterogeneous and mostly hypoechoic when compared with the adjacent subcutaneous soft tissues, although occasionally they appear isoechoic or hyperechoic (58) (Figure 9). On color Doppler US, VMs are slow flow lesions, with very low vascular density. Spectral analysis in the veins reveals low-velocity flow with non-modulated spectrum; sometimes flow is so slow that can hardly be detected. In these cases, compression of the lesion may help, as compression on vascular channels can augment outflow and release can increase inflow (Figure 9). On MRI, they demonstrate increased signal intensity on T2w images, which is even better visualized on fat-suppressed sequences like STIR (Figure 9). As they are not high flow lesions, they do not show flow-voids on T2w sequences (59). Valsalva maneuver can also be performed during MRI examination (Valsalva-augmented MRI). Due to their hemodynamic milieu and histological features, VMs can undergo thrombosis or hemorrhage, thus exhibiting a more variable signal intensity both on T1 and T2w images. Phleboliths are seen as high density and very-low-signal intensity foci at CT (Figure 9) and MR, respectively (Figure 9): they are visible in roughly half the cases (8), but when they are present they are virtually pathognomonic. On post-contrast images, VMs show gradual filling. They lack arterial and early venous enhancement and enlarged feeding vessels or arterio-venous shunting, thus differentiating them from high-flow lesions. Less often, they may demonstrate nodular enhancement of tortuous vessels on delayed venous phase image (8). On CT, VMs appear as lobulated or multilobulated lesions, isodense to muscle, that can cross fascial planes. Not only is CT useful in identifying phleboliths, but also in revealing bone remodeling. Dynamic CT acquisition during contrast administration, with Valsalva maneuver performed during the venous phase, can make the dilation readily apparent in the case of distensible lesions, especially when small. Diagnostic angiography is rarely necessary unless strong venous pulsation or monophasic continuous subtle bruits are present, suggesting a large vascular connection with the venous system. Phlebography by direct puncture can be performed prior to injecting any sclerosing agent to delineate the size of the multiple compartments of the malformation and the degree of communication with the venous system (56).

### Lymphatic malformation (LM)

Lymphatic malformations (LMs, previously known with the misnomer “lymphangiomas”; 5% of pediatric vascular and non-vascular orbital expansive lesions (60) are composed by a plexus of lymphatic channels (with or without a vascular component) that can form small (<2 cm) or large (>2 cm) chambers (61) and are classified into microcystic and macrocystic LMs, respectively, and “mixed”. Many authors suggest that in the orbit a diameter of 1 cm is a better threshold for discriminating between micro- and macro-cysts (62, 63) and are most commonly centered in the extra-conal compartment.

#### Clinical presentation

LMs of the orbit are present at birth, tend to grow with the patients and may present subacute signs and symptoms like progressive proptosis and globe displacement. Sometimes, LMs enlarge suddenly due to intralesional bleeding, venous thrombosis or proliferation of the internal lymphoid tissue (for example during a respiratory infection) and may present with abrupt pain, swelling, proptosis, ocular dysmotility or visual loss.

#### Imaging

**Figure 10:**
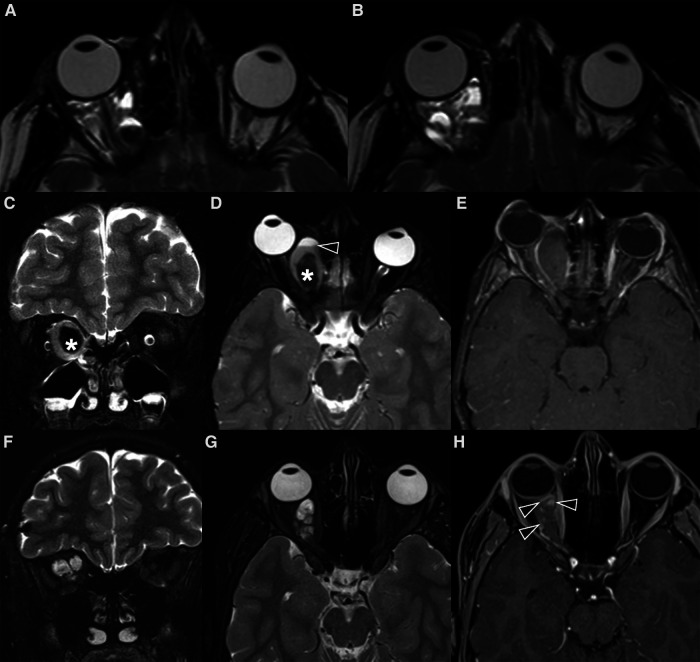
Imaging of lymphatic malformations. (**A,B**) MRI of a 11-year-old boy with subacute disturbances in visual motility and subtle ptoptosis of the right eye. Axial T2 weighted images show a right intraorbital multiloculated, polylobate and poorly defined mass with fluid-fluid levels, which extends into the extra and intra-conal compartments. The findings were virtually pathognomonic of a lymphatic malformation. (**C–H**) MRI of a 6-year-old girl with acute right orbital swelling and proptosis. (**C–E**) Coronal STIR (**C**) and axial T2 weighted images (**D**) show a multicompartmental expansile mass with marked compression on the optic nerve and proptosis; the lesion also demonstrates a central marked hypointensity (asterisk in **C,D**) with no evidence of enhancement on axial post-contrast fat-sat T1 (**E**). The mass was partially removed surgically, and histology revealed veno-lymphatic malformation. (**F-H**) 10-year follow-up MR images show how the mass has markedly reduced in size. The multiloculated architecture is more apparent (**F,G**), with thin intervening septa and blood-fluid levels; on post-contrast T1-w sequence only the septa enhance (**H**).

On gray-scale US, LMs appear as ill-defined and mostly anechoic lesions; echogenicity can be more heterogenous in the setting of recent bleeding or infection, even with the formation of possible fluid-fluid levels. The macrocystic lymphatic malformations appear as lesions containing numerous cystic formations of variable dimensions with liquid content separated by thin hyperechogenic septa (64); the lesion is deformable, and compression with the probe alters the shape of the cysts that never collapse completely (65). The microcystic types most often appear as solid hyperechoic formations. At Doppler US, presence of vascular signal is uncommon, as they are slow flow lesions (53) however in some cases it is possible to identify arterial vessels with high resistance confined in the larger septa (64). Owing to their non-encapsulated nature, they tend to violate fascial planes showing a trans-spatial growth pattern, often involving more than one anatomical compartment and being locally aggressive, engulfing and encasing vital structures: for this reason, the use of MR or CT is mandatory to assess the complete extension of the lesion, where MR is most preferable. LMs are usually seen on CT and MRI as poorly circumscribed, lobulated, trans-spatial lesions made up of numerous cystic spaces with intervening septations (Figure 10). The T1 and T2 signal intensity of the lesion depends on the presence and the temporal evolution of blood products: the cystic components are usually iso to slightly hyperintense on T1w imaging and very hyperintense on T2w imaging, with internal septations showing low signal intensity on T2; fluid-blood levels or blood-blood levels can be seen (Figure 10), usually indicating recent intralesional bleeding. After contrast administration, a subtle enhancement of the periphery and the septations may be demonstrated (Figure 10). On the other hand, in LM complicated by infection walls can become visibly thickened and show contrast enhancement (66).

## High-flow vascular malformations

### Artero-venous malformations (AVMs)

Arteriovenous malformations (AVMs) are defined by the presence of an abnormal vascular network (nidus) connecting the arterial and venous system, without an intervening normal capillary bed. They are rare and even rarer in the extra-cranial compartments, although they are the most frequent high-flow vascular malformations in the head and neck region accounting for 70% of high-flow lesions.

#### Clinical presentation

AVMs are always present at birth. Signs and symptoms of the AVMs vary depending on location, degree of arteriovenous shunting, venous hypertension (56), stage of the disease and age of the patient at presentation. Redness, pain and swelling around the affected eye – usually worse in the morning with some improvement during the day – are common presentation symptoms. Exophthalmos with or without pulsations, dilated corkscrew epibulbar vessels, swelling of the eyelids and engorged retinal veins may be also present. Deeper lesions may not always be clinically evident, and hemorrhage can be the only presenting symptoms. Functional complications – the most dreadful is visual deterioration – may ensue as a consequence of optic nerve or retinal ischemia, increased ocular pressure, shunting of arterial blood, mass effect or even when AVMs are approached incorrectly (surgically or endovascularly) and may react with an explosive growth (67).

The clinical evolution of AVMs is described by the Schöbinger classification (39):

• Stage I AVMs: simple skin staining with redness and warmth. With time they will progressively grow
• Stage II: pulsations, bruits and tortuous dilated veins become apparent.
• Stage III: local complications such as ulceration, bleeding, infection and pain.
• Stage IV: general complications. The AVM will produce a flow-steal syndrome with chronic fatigue and heart failure that may eventually be fatal.

#### Imaging

**Figure 11:**
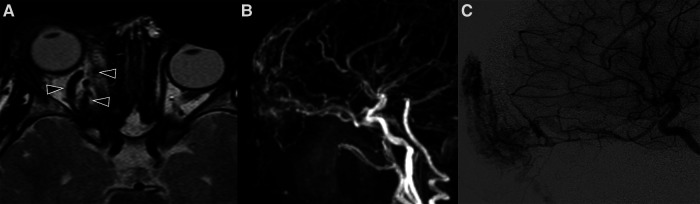
Mr and DSA of a 2-year-old boy referred for follow-up of an orbital “hemangioma” treated with propranolol and worsening of proptosis. Axial T2 TSE sequence (**A**) shows markedly dilated and tortuous vessels (arrowheads) in the extra- and intra-conal compartments of the right orbit without a discrete parenchymatous component, raising the suspicion of an AVM. MR angiography revealed a tangle of vessels with a small nidus and early venous drainage (**B**). The findings were subsequently confirmed at DSA (**C**).

**Figure 12:**
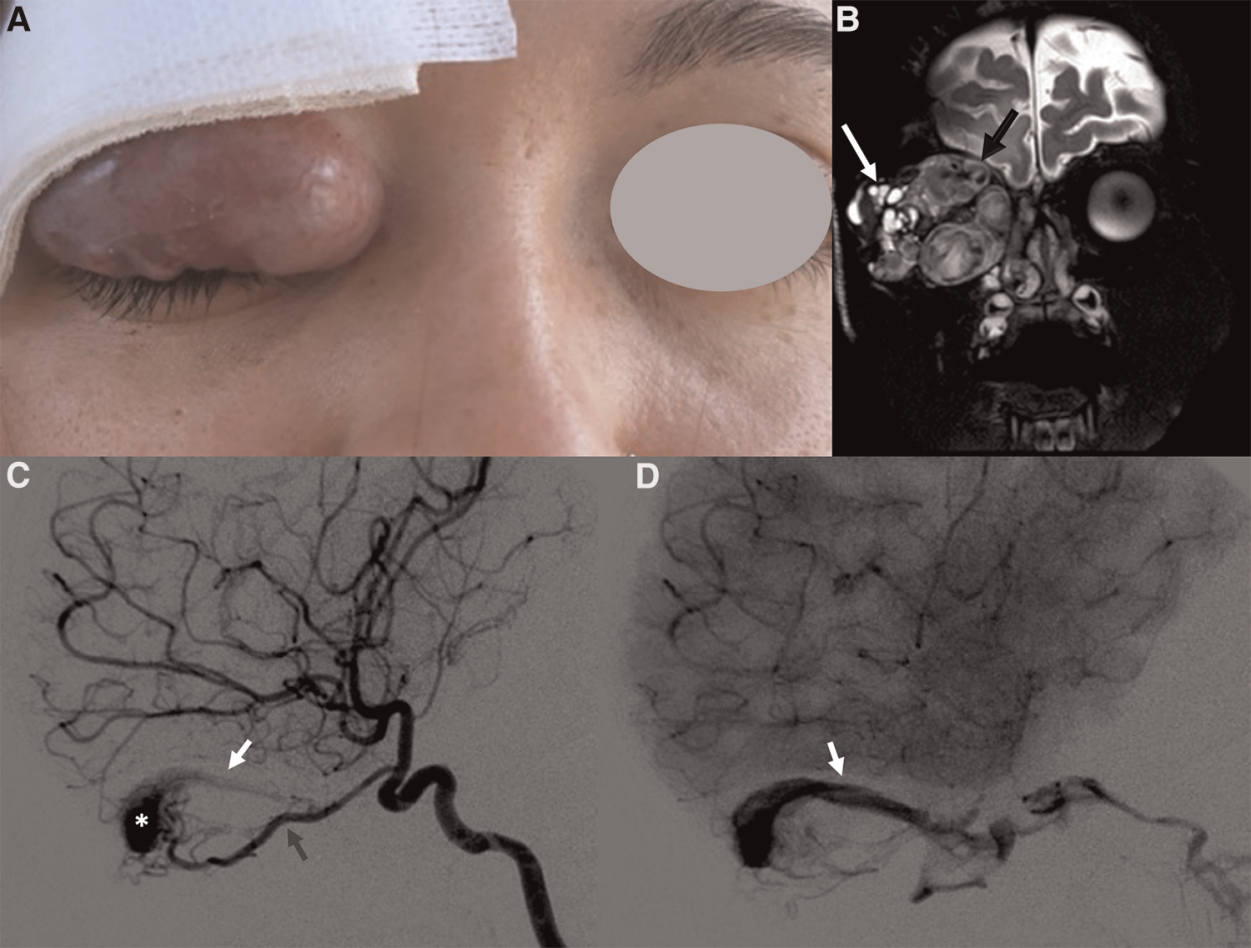
Right orbital AVM in a 14-year-old girl with progressive proptosis, strabismus and visual loss. A red pulsatile mass in the upper lid was already evident at clinical examination (**A**); a thrill was also perceptible at palpation. Coronal T2 TSE sequence (**B**) shows a massive trans-spatial expansile lesion occupying the entire orbit, distorting its anatomy; signal intensity is highly inhomogeneous due to the presence of tortuous flow-voids (black arrow in **B**) and blood products of varying age (white arrow in **B**). The other MR sequences did not demonstrate any intracranial extension of the vascular malformation (not shown). The arterial phase of the DSA (lateral view, **C**) depicts the dilated and slightly tortuous right ophthalmic artery (grey arrowhead, arising from the supraclinoid segment of the internal carotid artery) feeding the orbital AVM, the nidus (white asterisk) and the early opacification of the draining vein, the superior ophthalmic vein (white arrowhead). The venous drainage is better seen in the venous phase of the DSA (lateral view, **D**), occurring mainly through the dilated right superior ophthalmic vein (white arrowhead), which drains into the cavernous sinus.

Noninvasive imaging is mandatory: it allows proper confirmation of diagnosis, sets a baseline for the patient and helps define the architecture of the malformation (68). The AVM has no parenchymal component and therefore it appears on gray-scale US as a poorly defined heterogeneous structure, usually surrounded by fat. At the color Doppler examination, the malformation is a high flow lesion: there are numerous vessels (“high vascular density”) with increased diastolic flow and arterialization of the draining vein. The spectral Doppler analysis shows arterial vessels with high-velocity flow and low resistive index (64). MRI and CT angiography are ideal noninvasive modalities for visualizing the entire course and extent of an AVM, to assess organ involvement and for planning endovascular or surgical management (13). MRI is the finest technique to estimate the expansion of the malformation and its rapports with adjacent structures (69). Dilated and tortuous vascular structures representing the hypertrophied feeding arteries and the draining veins are seen, appearing as prominent flow voids on spin-echo sequences, in the absence of a discrete enhancing soft tissue mass, thus aiding in the differential diagnosis with hemangiomas (Figure 11). In the very early cases, subtly hypertrophied vessels can be the only findings visible (Figure 11). In case of hemorrhage or thrombosis, foci of hypersignal on T1w images will be found (Figure 12). When small, the AVM usually involves a single compartment whereas in larger lesions or those that have been previously treated the nidus may be difficult to define as pronounced secondarily induced angiogenesis is present (56) or may be centered in more than one compartment. At MRA there is rapid enhancement of the tangle of vessels, with early enhancement of the draining veins (Figure 12). Intraosseous infiltration causes low marrow signal intensity on T1-weighted images (70). The introduction of time-resolved MRI sequences allowed to evaluate the hemodynamics of an AVM, giving the precise topography, mapped in time, of arterial feeders and venous drainage prior to DSA: the progressive opacification of the nidus and of the draining veins can be well-demonstrated and critical findings such as arterial or intranidal aneurysms or venous varices can be easily visualized (Figures 11, 12). In children, the identification or documentation of an AVM in the cerebro-facial region should prompt investigation for other clinically silent AVMs along the same metameric level due to possible syndromic associations (71). In case of suspected bone involvement or when the AVM is centered within the bone, CT and CTA may be the best imaging tools. Intraosseous AVMs present as osteolytic lesions with intense enhancement, as the lytic bony defects represent dilated intraosseous venous pouches or dilated draining veins (56). CT may also show complications such as bone thickening (or mature periosteal reaction), seen as a result of chronic venous hypertension, or osteolysis. DSA remains the most invaluable tool to confirm the diagnosis: it delineates the angioarchitecture and real-time hemodynamics of the AVMs with no venous contamination of the arterial phase, allowing also their endovascular treatment when necessary (Figures 11, 12).

Like other vascular anomalies, AVMs can occur in isolation or as part of syndromes. Endothelial cells of the cephalic region have a regionalized origin from the paraxial mesoderm, which will finally provide the blood vessels to specific regions of the face and brain in a metameric fashion (72–74). Accordingly, the neural crest and mesodermal cells that originate from a given transverse level will be involved in the myogenesis and vasculogenesis of the corresponding facial territory: a somatic mutation in the neural crest prior to migration is thought to give rise to the non-hereditary diseases grouped under the term cerebro-facial arteriovenous metameric syndromes (CAMS) (75, 76). In their review, Bhattacharya and colleagues proposed three subtypes (CAMS I–III) based on involvement of the prosencephalon (medial or lateral) or rhombencephalon and thus lesion distribution in the cranio-facial region. Particularly, CAMS II (also known as Wyburn–Masson syndrome) derives from the lateral prosencephalic group with AVMs located along the optic pathway (from the optic nerve to the occipital lobe), in the thalamus and in the maxilla. While the intracranial counterparts are often clinically silent and rarely present with a neurologic deficit, intracranial hemorrhage or seizure, it is the orbital component of CAMS that leads to the diagnosis as the most common symptom is progressive visual loss (71).

## Differential diagnosis

Vascular anomalies of the orbit must be distinguished from other lesions sharing common clinical and radiological features. Indeed, the risk of high morbidity in such a complex anatomical location from mismanagement requires a systemic and multidisciplinary approach to facilitate an accurate diagnosis between the different entities based on clinical and imaging examination. Table 1 summarizes the main clinical and radiological features of the vascular lesions of the orbit.

**Table 1:** Clinical and radiological features of the principal vascular anomalies.

Diagnosis	Skin appearance	Palpation	Doppler ultrasound	MRI
Infantile hemangioma (IH)	Bright red plaque or tumor	Soft, compressible	well-demarcated variable echogenicity high vascularity: - high flow velocities during the proliferative phase - lower flow velocity during the involuting and involuted phases	T2/STIR hyperintense (variable amount of fat depending on the phase → T1w hyper) well-defined and lobulated (thin septa: T2w hypo) Flow voids on T2w Contrast enhancement: homogeneous (proliferative) or variable (involuting and involuted)
Congenital hemangioma (CH)	Red purple plaque or lump	Pasty consistency	See IH	See IH
Tufted angioma/kaposiform hemangioendothelioma	Red purple plaque or lump	Tender to tense (in case of KMP)	Superficial, heterogeneously echoic lesions with ill-defined margins	Non-specific
Capillary malformation	Homogeneous red patch from pink to port wine color	Not palpable	Usually confined to the dermis, showing occasionally Doppler flow	Subtle signal intensity abnormalities within the cutaneous or subcutaneous tissues or simply as skin thickening +/− contrast enhancement
Venous malformation	Blue patch or plaque	Soft, compressible with a slow refill	compressible, well-marginated spongiform appearance “slow flow pattern”	Variable T2w/T1w signal (usually T2 hyper) related to thrombosis, hemorrhage, phleboliths gradual filling is observed
Lymphatic malformation	Small grouped vesicles, purple skin in case of bleeding	Soft lump, tense in case of inflammation	Deformable numerous cystic formations of variable size vascular signal: uncommon	Trans-spatial and lobulated numerous cystic spaces with intervening septations fluid-blood levels or blood-blood levels
Arteriovenous malformation	Pink to red patch or plaque, sometimes surrounded by dilated veins	Tense, warm to touch, thrill, bruit point	no parenchymal component “high flow patter”	Dilated and tortuous vascular structures (hypertrophied feeding arteries and draining veins) Rapid enhancement of the tangle of vessels, with early enhancement of the draining veins

With respect to imaging, the understanding of orbital anatomy and blood flow patterns and the knowledge on how to obtain the best spatial and contrast resolution by US and especially MRI permit confident evaluation of the subtle structures of the orbit and increase diagnostic accuracy (77).

**Figure 13:**
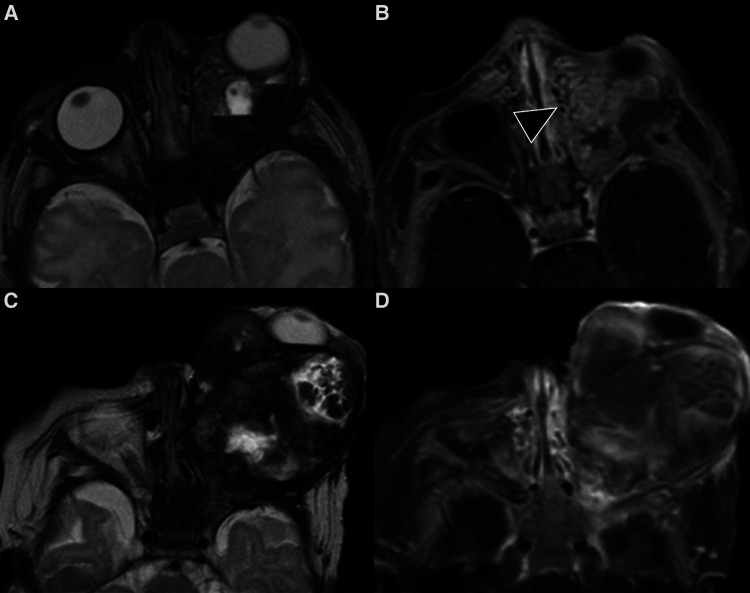
One-day-old boy with a congenital lesion of the left orbit. MR performed soon after birth shows a heterogeneous mass with multiple cystic-like components and fluid-blood levels on T2-weighted images (**A**) and a prominent solid component on axial T1 post-contrast images (black arrowhead in **B**); marked proptosis of the left eye is also evident. The lesion undergoes considerable growth after one month (**C,D**). The histological diagnosis was of rhabdomyosarcoma of the orbit.

However, it is worth recognizing that malignant lesions may mimic benign ones on standard MRI sequences. Although malignant lesions usually exhibit a low signal intensity on T2 due to their high cellularity, rhabdomyosarcoma may appear as a T2-bright lesion with well-defined margins and rarely presents with hemorrhagic or necrotic components, making it difficult to distinguish from a hemangioma. Adding to this, prominent flow-voids on TSE sequences – which were once thought to be pathognomonic of hemangiomas – in rare cases can also be found in rhabdomyosarcomas. Fortunately, the introduction of advanced MRI techniques in routine clinical practice allows a better functional characterization of lesional components than just morphological sequences. In their papers, Sepahdari and colleagues have reported that an ADC value of 1.0–1.15 × 10^−3^ mm^2^/s represents an optimal threshold for predicting malignancy with a sensitivity of 95%, specificity of 91% and accuracy of 93% (78, 79). The presence of intralesional hemorrhage may further complicate the diagnostic process. The sudden intralesional bleeding, whether within a benign or a malignant lesion, leads to the formation of fluid-fluid levels and oftentimes makes a malignant lesion completely indistinguishable from a lymphatic malformation: in this setting, the finding of an enhancing mass should raise the suspicion of a different entity (Figure 13).

**Figure 14:**
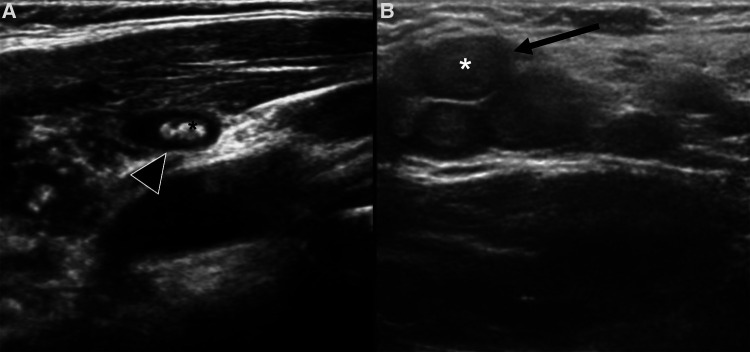
Different forms of target sign on US. (**A**) Phlebolith within a venous malformation, appearing as a hyperechoic structure (black asterisk) surrounded by an anechoic formation which represents the dilated venous channel (black arrowhead). (**B**) Plexiform neurofibromas appear as non-compressible, polylobate lesion made up of multiple confluent tubular masses (black arrow), consisting of a slightly hyperechoic core (asterisk) surrounded by a hypo/anechoic halo (black arrow).

The differential diagnosis of the masses of the orbit includes also lesions of neuronal origin. The “target sign” (Figure 14) is reported as typical of plexiform neurofibroma, but it is not specific and can also be seen in VMs (Figure 14). In the case of a plexiform neurofibroma, the typical appearance of the “target sign” (80) is that of a central component displaying low signal intensity on T2w images and subtle hyperechogenicity on US (corresponding at histopathology to central fibro-collagenous tissue) surrounded by a peripheral rim of high signal intensity on T2 weighted images and hypoechogenicity on US due to predominant myxoid tissue (81), while in venous malformations the central hypointense (T2w MRI) or hyperechoic (US) focus represents either a thrombus or a phlebolith in dilated venous channels (82) (Figure 14). Diffuse neurofibromas are less common, typically involve the skin and subcutaneous tissues, particularly of the head, and in MRI can mimic infantile and congenital hemangiomas (83).

However, the complexity of differential diagnoses demonstrate how – despite the existence of numerous diagnostic imaging methods – the radiological semiology of orbital lesions may not always be univocal and straightforward.

Adding to this, the radiological features do not always match with the clinical scenario. In these few cases, further invasive diagnostic modalities are the only way to establish an accurate diagnosis and to choose the appropriate management. Therefore, biopsy – excisional, incisional or fine needle aspiration – must be warranted for the rare cases in which a definitive diagnosis cannot be reached solely through clinical and radiological elements.

## Emerging treatments

The treatment of vascular anomalies is usually challenging. To date, only a few of them benefits from specific and curative therapies. The available treatments are medical, surgical, interventional, laser or combined: particularly, the fortuitous discovery that it was effective in regressing hemangiomas made the non-selective β-adrenergic receptor blocker propranolol a first-line therapy for these patients (84–87).

However, although they represent the standard of care, current therapeutic options are unfeasible in the majority of these patients due to the extensiveness of the malformations and high surgical morbidity, as well as to the disease progression and frequent recurrence.

Shedding light into the pathogenetic mechanism of most of the sporadic and familial vascular malformations, next generation sequencing disclosed how they share a common genetic mutational background with neoplasms. Indeed, the two major pathways involved are the RAS/ mitogen-activated protein kinase (MAPK)/extracellular signal-regulated kinase (ERK) and the phosphatidylinositol 3-kinase (PI3K)/protein kinase B (AKT)/mammalian target of rapamycin (mTOR) (88), both regulating cellular proliferation, migration and apoptosis (89).

These discoveries led to repurpose several target therapies available in oncology for the treatment of complex vascular malformations.

Rapamycin (or Sirolimus) has been the first developed targeting agent. Once reserved for tumors, this drug reduced the proliferation of endothelial cells and the growth of venous malformations in murine models (90) by inhibiting the mammalian Target of Rapamycin (mTOR). After preclinical studies, rapamycin demonstrated to be an efficacious and safety treatment of extensive or complex lymphatic and venous malformations (91, 92). Apart from mTOR inhibitors, other small molecules have been tested in vascular malformations: PIK3CA, AKT and MEK inhibitors in slow flow vascular malformations and BRAF and MEK inhibitors in fast flow vascular malformations. These new molecules are paving the way for new therapeutic perspectives based on precision medicine, but caution is needed since long term side effects are not yet known (89, 93).

## Discussion

Vascular anomalies are among the most frequent lesions of the pediatric orbit. The orbit relative small space and its complex anatomy pose specific considerations to be addressed, such as the difficulties to perform a biopsy, the high risk of complications both intrinsic to their evolution and relative to treatment that can lead to permanent functional deficits, not to mention the risk of cosmetic disfigurement. Thus, an early correct diagnosis allows an appropriate management. For this reason, pediatric patients with suspected vascular abnormalities should be evaluated by an expert multidisciplinary team including clinicians, radiologists, surgeons, pathologists, and geneticists. The role of the reference Centre is crucial for (i) diagnosis, (ii) patient follow-up, (iii) to guide and accompany the patient and family during the chronic and frequently disabling disorder, and (iv) to participate to research in the field, and (v) to recruit patients in clinical trial for emerging targeted therapies (3, 94).

Multimodal imaging is key in this process (19): US is non-invasive, rapid and low-cost, making it a very effective diagnostic method especially in children; together with Color Doppler US not only does it give many insights into internal architecture but also into vascularity of the lesion itself and can also help in narrowing the differential diagnosis (95, 96). MRI may be useful to showing the complete extension of the lesions as well as the degree of involvement of nearby structures. It should be performed on high field strengths scanners by means of head or orbital surface coils. Orbital surface coils obtain a higher signal-to-noise ratio than head coils, permitting the use of thinner sections and higher spatial resolution (97), while the use of head coils should be preferable when there is suspicion of a lesion that extends beyond the orbit.

In addition, some vascular syndromes are associated with other characteristic congenital anomalies, and affected patients have an increased risk of developing various malignancies. Patients with BWS are at increased risk of developing several embryonal malignancies including Wilms tumor, hepatoblastoma, rhabdomyosarcoma, and neuroblastoma (98, 99), while those with CLOVES (congenital lipomatous overgrowth, vascular malformations, epidermal nevi, and skeletal anomalies) have an increased risk for Wilms’ tumor (98). Genetic testing can be the key to perform appropriate diagnosis in syndromic patients (100): when a syndromic association is suspected (either on clinical grounds or at imaging sitting), the extension of the MRI study to other anatomical districts should be considered in order to look for associated abnormalities. Moreover, recognizing which phenotype is likely to be associated with somatic vs. germline mutations is fundamental to developing a thoughtful approach to genetic testing. In fact, the accurate identification of a genetic variant aids in confirming the diagnosis and in therapeutic decision making.

With new developing of new imaging techniques and endovascular treatment strategies not to mention artificial intelligence widely making is way, the role of radiologists will grow even more in the future.

## Conclusions

Vascular anomalies are among the most frequent lesions of the orbit in the pediatric population and necessarily require a multidisciplinary team evaluation for their adequate management, including clinicians, radiologists, surgeons and geneticists. Multimodality imaging allows their accurate classification into tumors or malformations according to the latest ISSVA classification and their anatomical definition in order to guide appropriate treatment. Moreover, the multidisciplinary team should be aware of the possibility that although the majority of these lesions are isolated, syndromic association do occur.
